# Supplementary material for: A Literature Review of Changes in Phase II Drug-Metabolizing Enzyme and Drug Transporter Expression during Pregnancy
Source: Pharmaceutics. 2023 Nov 15;15(11):2624. doi: 10.3390/pharmaceutics15112624 (PMC10674389; doi:10.3390/pharmaceutics15112624)
Supplement: Supplementary file 1 [file pharmaceutics-15-02624-s001.zip › SupplementaryMaterialS2.pdf]

Table S6. Summary of findings and implications for each phase II drug-metabolizing enzyme studied.

<table>
<tr><th>Enzyme</th><th>Results</th><th>Implications and/or Comments</th></tr>
<tr><td colspan="3">Overall, limited literature was found regarding phase II enzymes in pregnancy (i.e., enzyme expression/activity, pregnancy complications, hormonal regulation).
• The lack of literature found may suggest limitations of the search criteria, which could not encapsulate all relevant primary literature about the topic.
• Because animal data was not included, data analysis was limited to clinical data and in vitro data, with the assumption that observations in cell models translate to observations in humans unless evidenced otherwise.
• Further research is necessary to better understand the effect of pregnancy, pregnancy complications, and hormonal regulation on the expression/activity of phase II enzymes.</td></tr>
<tr><td>CMT</td><td>Placental CMT activity increased throughout the first trimester, achieved a peak at the beginning of the second trimester, then decreased until term [1].</td><td></td></tr>
<tr><td>COMT</td><td>Placental COMT activity was subject to interindividual variation at term [2].

Placental COMT protein expression was not significantly different between pregnancies complicated by preeclampsia and uncomplicated pregnancies [3,4].

17β-estradiol was observed to be a substrate of placental COMT [2].</td><td></td></tr>
<tr><td rowspan="2">GSTA</td><td>No significant change in maternal GSTA expression was observed throughout pregnancy [7], but an approximate 2-fold increase in enzyme expression was observed 6 weeks postpartum [6].

Pregnancies complicated by epilepsy [6], intrahepatic cholestasis [8,9], and preeclampsia [7] were associated with increased maternal GSTA protein expression.</td><td>Zusterzeel et al. provided no mechanistic hypothesis for the increase in enzyme expression observed 6 weeks postpartum [6].</td></tr>
<tr><td>A decrease in placental GSTA activity was observed in the first trimester [5].</td><td></td></tr>
<tr><td>GST-μ</td><td>No significant change in placental GST-μ activity or expression was observed during the first trimester [5] or in preeclamptic pregnancies [14].</td><td></td></tr>
<tr><td>GST-π</td><td>No significant change in placental GST-π activity was observed during the first trimester [5].

Placental GST-π activity was decreased in pregnancies complicated by diabetes [19], and placental GST-π protein expression was decreased in pregnancies complicated by preeclampsia [14].

Catecholamines were observed to downregulate placental GST-π activity [18].</td><td></td></tr>
</table>

**NAT1**	**Placental** NAT1 may increase during gestation [20].	
**NAT2**	**Maternal** NAT2 activity appeared to decrease in the first trimester, return to baseline in the second trimester, and remain at baseline in late pregnancy and postpartum [22,23].	While Tsutsumi et al. observed a statistically significant reduction (13%) in NAT2 activity, the reduction was not clinically significant [23].
**SULT1E1**	17β-estradiol was observed to be the preferred substrate for **placental** SULT1E1 [25].	
**UGT1A1**<br>**UGT1A3**<br>**UGT2B7**	Combined **maternal** UGT1A1, UGT1A3, and UGT2B7 activity appeared to increase from the first trimester through the third trimester, then appeared to return to baseline postdelivery [26].	No studies were found regarding individual changes in activity for the three enzymes. Considering the complex contribution of UGT1A1, UGT1A3, and UGT2B7 to buprenorphine metabolism, it is difficult to conclude which enzyme(s) are responsible for the changes in glucuronidation and, thus, which enzyme(s) exhibit altered activity during gestation. To deduce the metabolic mechanism behind gestational changes in indomethacin pharmacokinetics, Pillai et al. assessed the fractional contribution and gestational changes of each phase I and phase II enzyme in the indomethacin metabolism pathway [150], and a similar approach has been utilized to attempt to deduce the gestational changes in UGT activity for this case of buprenorphine glucuronidation.<br><br>Kinetics experiments conducted by Rouguieg et al. suggest that, in the buprenorphine glucuronidation pathway, UGT2B7 contributes to 41% and UGT1A1 contributes to 10% of glucuronidation, leaving 49% assumed to be attributed to UGT1A3 activity [144]. Because UGT1A1 is implied to have relatively minimal effect in this elimination pathway, the changes in enzyme activity observed in buprenorphine glucuronidation do not provide robust evidence to support changes in UGT1A1 activity.<br><br>On the contrary, a literature review conducted by van Hoogdalem et al. suggests that UGT1A1 is the major contributor to buprenorphine glucuronidation, followed by UGT2B7 and UGT1A3 [151]. The authors indicate that UGT1A1 and UGT2B7 activity are expected to increase during gestation, as supported by observed increases in area under the curve (AUC) ratios of buprenorphine glucuronide metabolite to parent drug.<br><br>Taken together, these data suggest that changes in the individual activity of UGT1A1, UGT1A3, and UGT2B7 cannot be determined by buprenorphine metabolism alone. Hence, these enzymes have been grouped together in the

		analysis, and the reported changes in activity for the individual enzymes represent their combined activity.
**UGT1A4**	Upregulation of **maternal** UGT1A4 mRNA expression and lamotrigine clearance was observed upon exposure of HepG2 cells to the levels of 17β-estradiol seen in pregnancy [28].	The upregulation of UGT1A4 expression by 17β-estradiol and the observed increase in substrate clearance support the contention that UGT1A4 activity may be increased during pregnancy, especially considering that the secretion of estrogens increases until term [143].<br><br>It should be noted that the upregulation of UGT1A4 expression observed in HepG2 cells was not observed in primary human hepatocytes, though the inconsistency between the two cell types is not unique to UGT1A4 [28].
	**Placental** UGT1A4 was present at term in placentas from pregnant women using lamotrigine and those from pregnant women not using lamotrigine [27].	
**UGT2B4**<br>**UGT2B7**<br>**UGT2B10**<br>**UGT2B11**<br>**UGT2B15**	**Placental** UGT2B4, UGT2B10, UGT2B11, and UGT2B15 were present at term [29].<br><br>**Placental** UGT2B7 was both present and subject to interindividual variation at term [29].	

Table S7. Summary of findings and implications for each drug transporter studied.

<table>
<tr><th>Transporter</th><th>Results</th><th>Implications and/or Comments</th></tr>
<tr><td colspan="3">Overall, limited pharmacokinetic data was found regarding drug transporters in the pregnant woman (i.e., transporter expression/activity, pregnancy complications, hormonal regulation).
The lack of literature found may suggest limitations of the search criteria, which could not encapsulate all relevant primary literature about the topic.
Because animal data was not included, data analysis was limited to clinical data and in vitro data, with the assumption that observations in cell models translate to observations in humans unless evidenced otherwise.
Further research is necessary to better understand the effect of pregnancy, pregnancy complications, and hormonal regulation on the expression/activity of drug transporters, especially considering the minimal or contradictory evidence that was found regarding the activity of certain transporters.
More literature was found regarding drug transporters in the placenta than drug transporters in the pregnant woman. Most of the evidence supporting placental transporter expression and activity was biological (i.e., mRNA expression, protein expression, intracellular accumulation of probe molecules), rather than pharmacokinetic.
The abundance of biological evidence may limit interpretation of the data, as we will assume that mRNA expression and protein expression translate to transporter activity unless evidenced otherwise. Where mRNA expression and protein expression demonstrate conflicting trends, changes in protein expression may be a more predictive indicator of changes in transporter activity.
While placental tissue (obtained following delivery or following termination of pregnancy) was largely utilized to study gestational changes in drug transporter activity, in vitro cell models (e.g., JAR, BeWo, transfected MDCKII) and trophoblast cultures were commonly utilized to study the effects of hormones on transporter activity.</td></tr>
<tr><td rowspan="2">P-gp</td><td>As suggested by pharmacokinetic data for maternal P-gp activity, renal P-gp activity was higher in the third trimester compared to postpartum [42], but intestinal P-gp activity did not change between the third trimester and postpartum [88].</td><td>In other words, following pregnancy, renal P-gp activity is suggested to decline to baseline [42].</td></tr>
<tr><td>Placental P-gp expression demonstrated peak activity in the first trimester, followed by a decrease through the third trimester [52-54,105].

Placentas from pregnancies complicated by chorioamnionitis [105], gestational diabetes [67], hepatitis C [136], human immunodeficiency virus [65], and preeclampsia [66] demonstrated changes in placental P-gp mRNA and/or protein expression compared to placentas from healthy pregnancies.

17β-estradiol and progesterone upregulated placental P-gp protein expression [63,64]. However, contradictory evidence suggests that progesterone inhibits P-gp efflux activity [37].</td><td>Regarding 17β-estradiol, data from placental JAR cells and isolated cytotrophoblasts agreed: P-gp mRNA expression and protein expression increased in both cell types [63,64]. Placental JAR cells and isolated cytotrophoblasts also demonstrated similar behavior when exposed to progesterone: P-gp mRNA expression was not observed to change, while P-gp protein expression was observed to increase. The inhibition of P-gp efflux activity following exposure to progesterone was observed in placental BeWo</td></tr>
</table>

		cells, which were used to model in vivo conditions, and the inconsistency between the upregulation of protein expression and the downregulation of efflux activity may suggest a complex interaction between progesterone and the placental P-gp transporter [37].
**MDR3**	Mutations in the MDR3 gene have been identified and correlated with intrahepatic cholestasis, though in a case study of one patient, the condition was not associated with a change in **maternal** MDR3 protein expression [93].	The studied patient was homozygous for SNP S320F in the MDR3 gene [93], and it is unclear whether this finding can be extrapolated to all pregnancies complicated by intrahepatic cholestasis.
	Conflicting data (i.e., increase or decrease) was found regarding **placental** MDR3 mRNA expression across gestation [92,94].<br><br>17β-estradiol increased **placental** MDR3 mRNA expression and protein expression [64].	Placental trophoblasts were treated with 100 nM 17β-estradiol, an attainable concentration during gestation [64].
**BSEP**	Mutations in the BSEP gene have been identified and correlated with intrahepatic cholestasis, and in a case study of one patient, the condition was associated with a decrease in **maternal** BSEP protein expression [93].<br><br>17β-estradiol decreased **hepatic** BSEP mRNA expression and protein expression [95].	The studied patient was homozygous for SNP V444A in the BSEP gene [93], and it is unclear whether this finding can be extrapolated to all pregnancies complicated by intrahepatic cholestasis.<br><br>Increasing levels of 17β-estradiol throughout pregnancy may decrease BSEP mRNA expression and protein expression, especially considering the concentration-dependent downregulation of BSEP by 17β-estradiol [95,143].
	**Placental** BSEP mRNA expression decreased from the first trimester to the third trimester [92,94]. Peak activity cannot be identified.	
**MRP3**	**Maternal** MRP3 mRNA expression and protein expression were upregulated by 17β-estradiol [102].	Hepatic LO2 cells were treated with 500 nM 17β-estradiol [102], a concentration that is higher than what is physiologically attainable (up to 50-100 nM 17β-estradiol and up to 10-500 nM progesterone) [63,64,143]; lower concentrations were not studied [102]. Increased levels of 17β-estradiol during pregnancy may increase MRP3 mRNA expression and protein expression [143], though more evidence is required to confirm changes in expression upon exposure to physiologically attainable concentrations of 17β-estradiol.
	**Placental** MRP3 mRNA expression increased from the first trimester to the third trimester [94]. Peak activity cannot be identified.	
**BCRP<br>OATP1B1<br>OATP1B3**	As suggested by pharmacokinetic data for **maternal** transporter activity, BCRP, OATP1B1, and/or OATP1B3 hepatic activity decreased from the third trimester through term, returning to baseline at postpartum [88].	In the study conducted by de Lima Moreira et al., pharmacokinetic parameters were measured following a single dose administration of Crestor® 5 mg to the same women in the third trimester and at term [88].

		Individual concentration vs. time curves were developed for each pregnant woman, and mean parameters were calculated.<br><br>No studies were found regarding individual changes in activity for the three enzymes. Considering the complex contribution of BCRP, OATP1B1, and OATP1B3 to rosuvastatin transport, it is difficult to conclude which transporter(s) are responsible for the increased exposure during the third trimester [88].<br><br>OATP1B1 and OATP1B3 were found to contribute more than 50% to the hepatic uptake of rosuvastatin, with OATP1B1-mediated rosuvastatin uptake observed to be significantly higher than OATP1B3-mediated rosuvastatin uptake [145]. Further, OATP1B3 was estimated to contribute 16-34% to rosuvastatin uptake, leaving 66-84% assumed to be attributable to OATP1B1 [146]. Considering the relatively minor role that OATP1B3 plays in rosuvastatin influx, the changes in enzyme activity observed in rosuvastatin metabolism do not provide robust evidence to support changes in OATP1B3 activity alone.<br><br>The contribution of BCRP to rosuvastatin efflux was studied in OATP1B1/BCRP double-transfected MDCKII cells, in which efflux clearance was 2.7-fold higher compared to OATP1B1 single-transfected cells [146]. Basal-to-apical efflux activity was also 1.7-fold higher than apical-to-basal influx activity, suggesting that the efflux activity of BCRP may have a greater contribution to rosuvastatin transport than the influx activity of OATP1B1. de Lima Moreira et al. report increased rosuvastatin exposure in third trimester women, which suggests decreased clearance and, likely, decreased efflux activity, considering the greater contribution of BCRP to rosuvastatin transport than OATP1B1 [88].<br><br>Because the fractional contribution of BCRP, OATP1B1, and OATP1B3 to rosuvastatin transport cannot be definitively deduced from the collected evidence, these transporters have been grouped together during the analysis, and the reported changes in activity for the individual transporters represent their combined activity.
	Conflicting data (i.e., increase, decrease, no change) was found regarding **placental** BCRP expression across gestation [80-82,105].	More evidence supported a decrease in BCRP protein expression at term [81,82,105], though one study supported an increase [80].

	Placentas from pregnancies complicated by chorioamnionitis [81], hepatitis C [136], human immunodeficiency virus [65], and preeclampsia [89] demonstrated changes in **placental** BCRP mRNA expression and/or protein expression.<br><br>17β-estradiol downregulated **placental** BCRP mRNA expression and protein expression, which was translated to decreased efflux activity [64,84]. Progesterone exhibited the opposite effect on BCRP expression and activity [84,86].	Additional evidence is required to confirm the change in BCRP protein expression in placentas affected by preeclampsia; while no significant change in protein expression was observed, an increasing trend was suggested [89].<br><br>Decreases in BCRP protein expression and efflux activity in response to 17β-estradiol were studied in BeWo cells [64], findings that were supported by the decrease in BCRP mRNA expression observed in placental explants [84]. The effects of progesterone on BCRP expression and activity were only studied in placental BeWo cells [84,86].
**OCT2<br>MATE1<br>MATE2**	As suggested by pharmacokinetic data for **maternal** transporter activity, OCT2, MATE1, and/or MATE2 activity increased from the first trimester to the second trimester and decreased from the third trimester to postpartum [113]. Activity between the second and third trimesters was inconclusive and depended on the probe (i.e., metformin or N1-methylnicotinamide) utilized.	No studies were found regarding individual changes in activity for the three enzymes. Due to the contribution of OCT2, MATE1, and MATE2 to metformin and N1-methylnicotinamide transport, it is difficult to conclude which transporter(s) are responsible for the changes in probe secretion clearance throughout pregnancy. Limited data is available regarding the individual contributions of OCT2, MATE1, and MATE2 to the vectorial transport of substrates [147].<br><br>In the kidney, OCT2 is believed to facilitate the basolateral uptake of cationic substrates, such as metformin, while MATE1 and MATE2 facilitate the coupled luminal secretion [148]. Uptake of metformin was significantly greater in OCT2-transfected MDCK cells compared to nontransfected MDCK cells, demonstrating the role of OCT2 as an uptake transporter [149]. Basal-to-apical transcellular transport of metformin was significantly greater in OCT2-MATE1-cotransfected MDCK cells compared to nontransfected MDCK cells and OCT2-transfected MDCK cells, demonstrating the role of MATE1 as an efflux transporter.<br><br>Considering the lack of evidence regarding individual contributions to transport and the coupled transport mechanism of OCT2, MATE1, and MATE2, similar gestational changes in enzyme activity have been assumed until more conclusive evidence suggests otherwise.<br><br>It should be noted that Bergagnini-Kolev et al. attributed transport-mediated changes in renal drug secretion solely to OCT2, MATE1, and MATE2 [113]. However, more recent knowledge suggests that OCT1, OCT3, and ENT4 also transport metformin [152], which further complicates interpretation of the data presented by Bergagnini-Kolev et al.

	A decrease in **placental** OCT2, MATE1, and MATE2 mRNA expression was observed from the first trimester to term [114]. Peak activity cannot be identified.	MATE1 mRNA expression was marginal in first trimester placentas and was not detected in term placentas [114]. Though this minimal decrease in expression demonstrated statistical significance, it does not provide sufficient evidence to support clinically relevant gestational changes in placental MATE1 expression, which have not been included in the analysis.<br><br>Results for MATE2 mRNA expression demonstrated a lack of statistical significance, but the clinical relevance of this decrease was not evaluated [114]. There is insufficient evidence to support gestational changes in placental MATE2 expression, which have not been included in the analysis.
**OAT1<br>OAT2<br>OAT3**	**Maternal** OAT1 activity was observed to decrease from the second trimester to postpartum [122]. Peak activity was estimated to occur in the second trimester, though further investigation is required to verify this conclusion.<br><br>**Maternal** OAT2 activity was observed to decrease from the third trimester to postpartum [122]. Peak activity was estimated to occur in the third trimester, though further investigation is required to verify this conclusion.<br><br>17β-estradiol and progesterone decreased OAT2 activity in transfected MDCK cells [123].<br><br>**Maternal** OAT3 activity demonstrated peak activity in the first trimester, followed by a decrease through the second trimester, third trimester, and postpartum [122].	In the experiments conducted by Ma et al., the concentrations that attained significant reductions in activity (1-100 μM 17β-estradiol and 10-100 μM progesterone) were higher than clinically attainable concentrations during pregnancy [123]. It is unclear whether the in vitro observations are representative of clinical findings for changes in OAT2 activity.
**CNT1<br>CNT2<br>CNT3**	**Placental** CNT1, CNT2, and CNT3 mRNA expression increased from the first trimester to term [128].	CNT1 mRNA expression was not detected in first trimester placentas and was marginal in term placentas, and this minimal increase in expression demonstrated a lack of significance [128]. There is insufficient evidence to support gestational changes in placental CNT1 expression, and thus, the transporter has not been included in the analysis.
**ENT1<br>ENT2**	In experiments to confirm the hepatic uptake of entecavir, 17β-estradiol and progesterone decreased ENT1 activity in transfected HEK293 cells [123].	In the experiments conducted by Ma et al., the concentrations that attained significant reductions in activity (5-100 μM 17β-estradiol and 50-100 μM progesterone) were higher than clinically attainable concentrations during pregnancy [123]. It is unclear whether the in vitro observations are representative of clinical findings for changes in ENT1 activity.
	There was no change in **placental** ENT1 or ENT2 mRNA expression from the first trimester to the third trimester [131].	

	Placentas from pregnancies complicated by gestational diabetes demonstrated decreased **placental** ENT1 and ENT2 protein expression, both of which were paralleled by reductions in transporter activity [137].<br><br>Placentas from pregnancies complicated by preeclampsia demonstrated decreased **placental** ENT1 protein expression but increased **placental** ENT2 protein expression, which were paralleled by respective changes in transporter activity [135].	
**OCT3**	Though **placental** OCT3 protein expression increased from the first trimester through the third trimester [54,116], OCT3 mRNA expression was reported to decrease from the first trimester to term [114].<br><br>Placentas from pregnancies complicated by human immunodeficiency virus demonstrated decreased **placental** OCT3 mRNA expression and protein expression [65].	Inconsistencies between protein expression and mRNA expression suggest that changes at the molecular level may not translate to changes at the protein level or activity level.
**MRP1**	Placentas from pregnancies complicated by human immunodeficiency virus demonstrated decreased **placental** MRP1 mRNA expression [65].<br><br>Progesterone upregulated **placental** MRP1 mRNA expression but had no effect on MRP1 protein expression [64].	No information was found regarding changes in maternal or placental MRP1 expression in uncomplicated pregnancies.<br><br>Placental trophoblasts were treated with 100 nM progesterone, an attainable concentration during gestation [64]. Due to inconsistencies in the effect of progesterone on MRP1 mRNA expression and protein expression, it has been assumed that the lack of change in MRP1 protein expression translates to a lack of change in MRP1 activity. However, considering that MRP1 protein expression was not evaluated at higher concentrations of progesterone, further evidence is necessary to confirm the lack of change across gestational stages.
**MRP2**	Conflicting data (i.e., increase or decrease) was found regarding **placental** MRP2 mRNA expression across gestation [94,100].<br><br>Changes in **placental** MRP2 mRNA expression were observed in placentas affected by human immunodeficiency virus [65] and intrahepatic cholestasis [101].	
**MRP4**	Decreased **placental** MRP4 mRNA expression was observed in placentas affected by human immunodeficiency virus [65].	
**MRP5**	A decrease in **placental** MRP5 mRNA expression was observed in both the second and third trimester [104]. Peak activity cannot be identified.	

**OATP1A2**	**Placental** OATP1A2 mRNA expression decreased from the first trimester to the third trimester [92]. Peak activity cannot be identified.<br><br>Decreased **placental** OATP1A2 mRNA expression and protein expression were observed in placentas affected by intrahepatic cholestasis [108].	
**OATP2B1**	While **placental** OATP2B1 protein abundance was observed to decrease between the first and second trimester [54], conflicting data (i.e., increase or decrease) was found regarding placental OATP2B1 mRNA expression across gestation [81,109].<br><br>Decreases in **placental** OATP2B1 mRNA expression were observed in placentas affected by chorioamnionitis [81] and human immunodeficiency virus [65].	If mRNA and protein expression have similar effects on transporter activity, second trimester protein abundance can be compared to term mRNA expression to describe gestational changes in placental transporter expression.<br><br>Additional evidence is required to confirm the change in OATP2B1 protein expression in placentas affected by chorioamnionitis; while no significant change in protein expression was observed, a decreasing trend was suggested [81].
**OATP3A1**	**Placental** OATP3A1 mRNA expression decreased from the first trimester to the third trimester [92]. Peak activity cannot be identified.	
**OATP4A1**	Increases in **placental** OATP4A1 mRNA expression were observed in placentas affected by human immunodeficiency virus [65].	
**OCT1**	Decreases in **placental** OCT1 mRNA expression were observed in placentas affected by human immunodeficiency virus [65].	
**OCTN2**	A decrease in **placental** OCTN2 mRNA expression, though not considered to be significant, was observed from the third trimester to term [119].	The lack of significant results for OCTN2 mRNA expression demonstrated insufficient evidence to support gestational changes in transporter expression from the second trimester to the third trimester [119]; from the available data, it can only be concluded that placental OCTN2 mRNA expression does not appear to change throughout the third trimester. Considering the importance of OCTN2 to placental carnitine transport, the lack of change in late pregnancy may indicate that changes occurred earlier in pregnancy.
**OAT4**	**Placental** OAT4 protein expression increased from the second trimester to the third trimester [54].<br><br>**Placental** OAT4 mRNA was downregulated in placentas complicated by human immunodeficiency virus [65].<br><br>**Placental** OAT4 activity was unaffected by 17β-estradiol but was downregulated by progesterone [126].	The effects of 17β-estradiol and progesterone on OAT4 activity were studied in OAT4-expressing BeWo cells [126]. Significant downregulation of OAT4

		activity was only observed at high concentrations of progesterone (10 μM); no change in OAT4 activity was observed at progesterone concentrations that are physiologically attainable during pregnancy, suggesting that OAT4 activity may not exhibit gestational changes.
**FcRn**	The increases in fetal IgG concentration and fetal-to-maternal IgG ratio with increasing gestational age suggest increased **placental** FcRn transport capacity throughout pregnancy [142].	To facilitate the increase in placental FcRn transport capacity, the expression or activity of the FcRn transporter likely increases with increasing gestational age.
**MRP7**<br>**MRP8**<br>**NTCP**<br>**MCT3**<br>**OATP2A1**<br>**OCTN1**<br>**ENT4**	No information regarding transporter expression/activity, pregnancy complications, or hormonal regulation were found.	
