# Supplementary material for: A Literature Review of Changes in Phase II Drug-Metabolizing Enzyme and Drug Transporter Expression during Pregnancy
Source: Pharmaceutics. 2023 Nov 15;15(11):2624. doi: 10.3390/pharmaceutics15112624 (PMC10674389; doi:10.3390/pharmaceutics15112624)
Supplement: Supplementary file 1 [file pharmaceutics-15-02624-s001.zip › SupplementaryMaterialS3.pdf]

Studies Included in Review

1. Paik, M.K.; Hwang, B.D.; Lim, K. Human Placenta S-Adenosylmethionine: Protein Carboxyl O-Methyltransferase (Protein Methylase II). Purification and Characterization. *Int. J. Biochem.* **1988**, *20*, 1107-1112.
2. Zhu, B.T.; Wu, K.Y.; Wang, P.; Cai, M.X.; Conney, A.H. O-Methylation of Catechol Estrogens by Human Placental Catechol-O-Methyltransferase: Interindividual Differences in Sensitivity to Heat Inactivation and to Inhibition by Dietary Polyphenols. *Drug Metab. Dispos.* **2010**, *38*, 1892-1899.
3. Seol, H.J.; Cho, G.J.; Oh, M.J.; Kim, H.J. 2-Methoxyoestradiol Levels and Placental Catechol-O-Methyltransferase Expression in Patients with Late-Onset Preeclampsia. *Arch. Gynecol. Obstet.* **2013**, *287*, 881-886.
4. Palmer, K.; Saglam, B.; Whitehead, C.; Stock, O.; Lappas, M.; Tong, S. Severe Early-Onset Preeclampsia is Not Associated with a Change in Placental Catechol O-Methyltransferase (COMT) Expression. *Am. J. Pathol.* **2011**, *178*, 2484-2488.
5. Datta, K.; Roy, S.K.; Mitra, A.K.; Kulkarni, A.P. Glutathione S-Transferase Mediated Detoxification and Bioactivation of Xenobiotics During Early Human Pregnancy. *Early Hum. Dev.* **1994**, *37*, 167-174.
6. Zusterzeel, P.L.; Knapen, M.F.; Roes, E.M.; Steegers-Theunissen, R.P.; Peters, W.H.; Merkus, H.M.; Steegers, E.A. Glutathione S-Transferase Alpha Levels in Epileptic and Healthy Women Preconceptionally and Throughout Pregnancy. *Gynecol. Obstet. Invest.* **1999**, *48*, 89-92.
7. Knapen, M.F.; Mulder, T.P.; Bisseling, J.G.; Penders, R.H.; Peters, W.H.; Steegers, E.A. Plasma Glutathione S-Transferase Alpha 1-1: A More Sensitive Marker for Hepatocellular Damage than Serum Alanine Aminotransferase in Hypertensive Disorders of Pregnancy. *Am. J. Obstet. Gynecol.* **1998**, *178*, 161-165.
8. Joutsiniemi, T.; Leino, R.; Timonen, S.; Pulkki, K.; Ekblad, U. Hepatocellular Enzyme Glutathione S-Transferase Alpha and Intrahepatic Cholestasis of Pregnancy. *Acta Obstet. Gynecol. Scand.* **2008**, *87*, 1280-1284.
9. Dann, A.T.; Kenyon, A.P.; Seed, P.T.; Poston, L.; Shennan, A.H.; Tribe, R.M. Glutathione S-Transferase and Liver Function in Intrahepatic Cholestasis of Pregnancy and Pruritus Gravidarum. *Hepatology* **2004**, *40*, 1406-1414.
10. Dalmizrak, O.; Kulaksiz-Erkmen, G.; Ozer, N. Fluoxetine-Induced Toxicity Results in Human Placental Glutathione S-Transferase-π (GST-π) Dysfunction. *Drug Chem. Toxicol.* **2016**, *39*, 439-444.
11. Dalmizrak, O.; Kulaksiz-Erkmen, G.; Ozer, N. The Inhibition Characteristics of Human Placental Glutathione S-Transferase-π by Tricyclic Antidepressants: Amitriptyline and Clomipramine. *Mol. Cell Biochem.* **2011**, *355*, 223-231.
12. Dalmizrak, O.; Kulaksiz-Erkmen, G.; Ozer, N. Possible Prenatal Impact of Sertraline on Human Placental Glutathione S-Transferase-π. *Hum. Exp. Toxicol.* **2012**, *31*, 457-464.
13. Dalmizrak, O.; Kulaksiz-Erkmen, G.; Ozer, N. Evaluation of the In Vitro Inhibitory Impact of Hypericin on Placental Glutathione S-Transferase Pi. *Protein J.* **2012**, *31*, 544-549.
14. Zusterzeel, P.L.; Peters, W.H.; De Bruyn, M.A.; Knapen, M.F.; Merkus, H.M.; Steegers, E.A. Glutathione S-Transferase Isoenzymes in Decidua and Placenta of Preeclamptic Pregnancies. *Obstet. Gynecol.* **1999**, *94*, 1033-1038.
15. Vander Jagt, D.L.; Wilson, S.P.; Heidrich, J.E. Purification and Bilirubin Binding Properties of Glutathione S-Transferase from Human Placenta. *FEBS Lett.* **1981**, *136*, 319-321.
16. McRobie, D.J.; Glover, D.D.; Tracy, T.S. Regiospecificity of Placental Metabolism by Cytochromes P450 and Glutathione S-Transferase. *Gynecol. Obstet. Invest.* **1996**, *42*, 154-158.
17. Polidoro, G.; di Ilio, C.; del Boccio, G.; Zulli, P.; Federici, G. Glutathione S-Transferase Activity in Human Placenta. *Biochem. Pharmacol.* **1980**, *29*, 1677-1680.
18. Barszczewska, I.; Barańczyk-Kuźma, A. Placenta as a Protecting Barrier--Some Properties of Glutathione-S-Transferase. *Acta Biochim. Pol.* **1992**, *39*, 147-152.
19. McRobie, D.J.; Glover, D.D.; Tracy, T.S. Effects of Gestational and Overt Diabetes on Human Placental Cytochromes P450 and Glutathione S-Transferase. *Drug Metab. Dispos.* **1998**, *26*, 367-371.

20. Derewlany, L.O.; Knie, B.; Koren, G. Arylamine N-Acetyltransferase Activity of the Human Placenta. *J. Pharmacol. Exp. Ther.* **1994**, *269*, 756-760.
21. Smelt, V.A.; Mardon, H.J.; Sim, E. Placental Expression of Arylamine N-Acetyltransferases: Evidence for Linkage Disequilibrium Between NAT1*10 and NAT2*4 Alleles of the Two Human Arylamine N-Acetyltransferase Loci NAT1 and NAT2. *Pharmacol. Toxicol.* **1998**, *83*, 149-157.
22. Han, L.W.; Ryu, R.J.; Cusumano, M.; Easterling, T.R.; Phillips, B.R.; Risler, L.J.; Shen, D.D.; Hebert, M.F. Effect of N-Acetyltransferase 2 Genotype on the Pharmacokinetics of Hydralazine During Pregnancy. *J. Clin. Pharmacol.* **2019**, *59*, 1678-1689.
23. Tsutsumi, K.; Kotegawa, T.; Matsuki, S.; Tanaka, Y.; Ishii, Y.; Kodama, Y.; Kuranari, M.; Miyakawa, I.; Nakano, S. The Effect of Pregnancy on Cytochrome P4501A2, Xanthine Oxidase, and N-Acetyltransferase Activities in Humans. *Clin. Pharmacol. Ther.* **2001**, *70*, 121-125.
24. Bernier, F.; Lopez-Solache, I.; Labrie, F.; Luu-The, V. Cloning and Expression of cDNA Encoding Human Placental Estrogen Sulfotransferase. *Mol. Cell. Endocrinol.* **1994**, *99*, R11-R15.
25. Tseng, L.; Lee, L.Y.; Mazella, J. Estrogen Sulfotransferase in Human Placenta. *J. Steroid Biochem.* **1985**, *22*, 611-615.
26. Zhang, H.; Bastian, J.R.; Zhao, W.; Chen, H.; Shaik, I.H.; Chaphekar, N.; Caritis, S.N.; Venkataramanan, R. Pregnancy Alters CYP- and UGT-Mediated Metabolism of Buprenorphine. *Ther. Drug Monit.* **2020**, *42*, 264-270.
27. Reimers, A.; Østby, L.; Stuen, I.; Sundby, E. Expression of UDP-Glucuronosyltransferase 1A4 in Human Placenta at Term. *Eur. J. Drug Metab. Pharmacokinet.* **2011**, *35*, 79-82.
28. Chen, H.; Yang, K.; Choi, S.; Fischer, J.H.; Jeong, H. Up-regulation of UDP-Glucuronosyltransferase (UGT) 1A4 by 17Beta-Estradiol: A Potential Mechanism of Increased Lamotrigine Elimination in Pregnancy. *Drug Metab. Dispos.* **2009**, *37*, 1841-1847.
29. Collier, A.C.; Ganley, N.A.; Tingle, M.D.; Blumenstein, M.; Marvin, K.W.; Paxton, J.W.; Mitchell, M.D.; Keelan, J.A. UDP-Glucuronosyltransferase Activity, Expression and Cellular Localization in Human Placenta at Term. *Biochem. Pharmacol.* **2002**, *63*, 409-419.
30. Tupova, L.; Hirschmugl, B.; Sucha, S.; Pilarova, V.; Székely, V.; Bakos, E.; Novakova, L.; Özvegy-Laczka, C.; Wadsack, C.; Ceckova, M. Interplay of Drug Transporters P-Glycoprotein (MDR1), MRP1, OATP1A2 and OATP1B3 in Passage of Maraviroc Across Human Placenta. *Biomed. Pharmacother.* **2020**, *129*, 110506.
31. Bai, M.; Ma, Z.; Sun, D.; Zheng, C.; Weng, Y.; Yang, X.; Jiang, T.; Jiang, H. Multiple Drug Transporters Mediate the Placental Transport of Sulpiride. *Arch. Toxicol.* **2017**, *91*, 3873-3884.
32. Ma, Z.; Yang, X.; Jiang, T.; Bai, M.; Zheng, C.; Zeng, S.; Sun, D.; Jiang, H. Multiple SLC and ABC Transporters Contribute to the Placental Transfer of Entecavir. *Drug Metab. Dispos.* **2017**, *45*, 269-278.
33. Afrouzian, M.; Al-Lahham, R.; Patrikeeva, S.; Xu, M.; Fokina, V.; Fischer, W.; Abdel-Rahman, S.; Constantine, M.; Ahmed, M.; Nanovskaya, T. Role of the Efflux Transporters BCRP and MRP1 in Human Placental Bio-Disposition of Pravastatin. *Biochem. Pharmacol.* **2018**, *156*, 467-478.
34. Neumanova, Z.; Cerveny, L.; Greenwood, S.L.; Ceckova, M.; Staud, F. Effect of Drug Efflux Transporters on Placental Transport of Antiretroviral Agent Abacavir. *Reprod. Toxicol.* **2015**, *57*, 176-182.
35. Nakamura, Y.; Ikeda, S.; Furukawa, T.; Sumizawa, T.; Tani, A.; Akiyama, S.; Nagata, Y. Function of P-Glycoprotein Expressed in Placenta and Mole. *Biochem. Biophys. Res. Commun.* **1997,** *235*, 849-853.
36. Sudhakaran, S.; Rayner, C.R.; Li, J.; Kong, D.C.; Gude, N.M.; Nation, R.L. Inhibition of Placental P-Glycoprotein: Impact on Indinavir Transfer to the Foetus. *Br. J. Clin. Pharmacol.* **2008**, *65*, 667-673.
37. Ushigome, F.; Takanaga, H.; Matsuo, H.; Yanai, S.; Tsukimori, K.; Nakano, H.; Uchiumi, T.; Nakamura, T.; Kuwano, M.; Ohtani, H.; Sawada, Y. Human Placental Transport of Vinblastine, Vincristine, Digoxin and Progesterone: Contribution of P-Glycoprotein. *Eur. J. Pharmacol.* **2000**, *408*, 1-10.
38. Pinto, L.; Bapat, P.; de Lima Moreira, F.; Lubetsky, A.; de Carvalho Cavalli, R.; Berger, H.; Lanchote, V.R.; Koren, G. Chiral Transplacental Pharmacokinetics of Fexofenadine: Impact of P-Glycoprotein Inhibitor Fluoxetine Using the Human Placental Perfusion Model. *Pharm. Res.* **2021**, *38*, 647-655.
39. Rahi, M.; Heikkinen, T.; Härtter, S.; Hakkola, J.; Hakala, K.; Wallerman, O.; Wadelius, M.; Wadelius, C.; Laine, K. Placental Transfer of Quetiapine in Relation to P-Glycoprotein Activity. *J. Psychopharmacol.* **2007**, *21*, 751-756.

40. Hemauer, S.J.; Patrikeeva, S.L.; Wang, X.; Abdelrahman, D.R.; Hankins, G.D.; Ahmed, M.S.; Nanovskaya, T.N. Role of Transporter-Mediated Efflux in the Placental Biodisposition of Bupropion and Its Metabolite, OH-Bupropion. *Biochem. Pharmacol.* **2010**, *80*, 1080-1086.
41. Nekhayeva, I.A.; Nanovskaya, T.N.; Hankins, G.D.; Ahmed, M.S. Role of Human Placental Efflux Transporter P-Glycoprotein in the Transfer of Buprenorphine, Levo-Alpha-Acetylmethadol, and Paclitaxel. *Am. J. Perinatol.* **2006**, *23*, 423-430.
42. Hebert, M.F.; Easterling, T.R.; Kirby, B.; Carr, D.B.; Buchanan, M.L.; Rutherford, T.; Thummel, K.E.; Fishbein, D.P.; Unadkat, J.D. Effects of Pregnancy on CYP3A and P-Glycoprotein Activities as Measured by Disposition of Midazolam and Digoxin: A University of Washington Specialized Center of Research Study. *Clin. Pharmacol. Ther.* **2008**, *84*, 248-253.
43. Hemauer, S.J.; Patrikeeva, S.L.; Nanovskaya, T.N.; Hankins, G.D.; Ahmed, M.S. Role of Human Placental Apical Membrane Transporters in the Efflux of Glyburide, Rosiglitazone, and Metformin. *Am. J. Obstet. Gynecol.* **2010**, *202*, 383.e1-383.e3837.
44. Hemauer, S.J.; Patrikeeva, S.L.; Nanovskaya, T.N.; Hankins, G.D.; Ahmed, M.S. Opiates Inhibit Paclitaxel Uptake by P-Glycoprotein in Preparations of Human Placental Inside-Out Vesicles. *Biochem. Pharmacol.* **2009**, *78*, 1272-1278.
45. Holcberg, G.; Sapir, O.; Tsadkin, M.; Huleihel, M.; Lazer, S.; Katz, M.; Mazor, M.; Ben-Zvi, Z. Lack of Interaction of Digoxin and P-Glycoprotein Inhibitors, Quinidine and Verapamil in Human Placenta In Vitro. *Eur. J. Obstet. Gynecol. Reprod. Biol.* **2003**, *109*, 133-137.
46. Neumanova, Z.; Cerveny, L.; Ceckova, M.; Staud, F. Interactions of Tenofovir and Tenofovir Disoproxil Fumarate with Drug Efflux Transporters ABCB1, ABCG2, and ABCC2; Role in Transport Across the Placenta. *AIDS* **2014**, *28*, 9-17.
47. Vinot, C.; Gavard, L.; Tréluyer, J.M.; Manceau, S.; Courbon, E.; Scherrmann, J.M.; Declèves, X.; Duro, D.; Peytavin, G.; Mandelbrot, L.; Giraud, C. Placental Transfer of Maraviroc in an Ex Vivo Human Cotyledon Perfusion Model and Influence of ABC Transporter Expression. *Antimicrob. Agents Chemother.* **2013**, *57*, 1415-1420.
48. Rahi, M.; Heikkinen, T.; Hakkola, J.; Hakala, K.; Wallerman, O.; Wadelius, M.; Wadelius, C.; Laine, K. Influence of Adenosine Triphosphate and ABCB1 (MDR1) Genotype on the P-Glycoprotein-Dependent Transfer of Saquinavir in the Dually Perfused Human Placenta. *Hum. Exp. Toxicol.* **2008**, *27*, 65-71.
49. Mölsä, M.; Heikkinen, T.; Hakkola, J.; Hakala, K.; Wallerman, O.; Wadelius, M.; Wadelius, C.; Laine, K. Functional Role of P-Glycoprotein in the Human Blood-Placental Barrier. *Clin. Pharmacol. Ther.* **2005**, *78*, 123-131.
50. Mark, P.J.; Waddell, B.J. P-Glycoprotein Restricts Access of Cortisol and Dexamethasone to the Glucocorticoid Receptor in Placental BeWo Cells. *Endocrinology* **2006**, *147*, 5147-5152.
51. Ushigome, F.; Koyabu, N.; Satoh, S.; Tsukimori, K.; Nakano, H.; Nakamura, T.; Uchiumi, T.; Kuwano, M.; Ohtani, H.; Sawada, Y. Kinetic Analysis of P-Glycoprotein-Mediated Transport by Using Normal Human Placental Brush-Border Membrane Vesicles. *Pharm. Res.* **2003**, *20*, 38-44.
52. Gil, S.; Saura, R.; Forestier, F.; Farinotti, R. P-Glycoprotein Expression of the Human Placenta During Pregnancy. *Placenta* **2005**, *26*, 268-270.
53. Sun, M.; Kingdom, J.; Baczyk, D.; Lye, S.J.; Matthews, S.G.; Gibb, W. Expression of the Multidrug Resistance P-Glycoprotein, (ABCB1 Glycoprotein) in the Human Placenta Decreases with Advancing Gestation. *Placenta* **2006**, *27*, 602-609.
54. Anoshchenko, O.; Prasad, B.; Neradugomma, N.K.; Wang, J.; Mao, Q.; Unadkat, J.D. Gestational Age-Dependent Abundance of Human Placental Transporters as Determined by Quantitative Targeted Proteomics. *Drug Metab. Dispos.* **2020**, *48*, 735-741.
55. MacFarland, A.; Abramovich, D.R.; Ewen, S.W.; Pearson, C.K. Stage-Specific Distribution of P-Glycoprotein in First-Trimester and Full-Term Human Placenta. *Histochem J.* **1994**, *26*, 417-423.
56. Kozłowska-Rup, D.; Czekaj, P.; Plewka, D.; Sikora, J. Immunolocalization of ABC Drug Transporters in Human Placenta from Normal and Gestational Diabetic Pregnancies. *Ginekol. Pol.* **2014**, *85*, 410-419.
57. Nagashige, M.; Ushigome, F.; Koyabu, N.; Hirata, K.; Kawabuchi, M.; Hirakawa, T.; Satoh, S.; Tsukimori, K.; Uchiumi, T.; Kuwano, M.; Ohtani, H.; Sawada, Y. Basal Membrane Localization of MRP1 in Human Placental Trophoblast. *Placenta* **2003**, *24*, 951-958.

58. Mylona, P.; Glazier, J.D.; Greenwood, S.L.; Sides, M.K.; Sibley, C.P. Expression of the Cystic Fibrosis (CF) and Multidrug Resistance (MDR1) Genes During Development and Differentiation in the Human Placenta. *Mol. Hum. Reprod.* **1996**, *2*, 693-698.
59. Mylona, P.; Hoyland, J.A.; Sibley, C.P. Sites of mRNA Expression of the Cystic Fibrosis (CF) and Multidrug Resistance (MDR1) Genes in the Human Placenta of Early Pregnancy: No Evidence for Complementary Expression. *Placenta* **1999**, *20*, 493-496.
60. Hodyl, N.A.; Stark, M.J.; Butler, M.; Clifton, V.L. Placental P-Glycoprotein is Unaffected by Timing of Antenatal Glucocorticoid Therapy but Reduced in SGA Preterm Infants. *Placenta* **2013**, *34*, 325-330.
61. Lye, P.; Bloise, E.; Nadeem, L.; Gibb, W.; Lye, S.J.; Matthews, S.G. Glucocorticoids Modulate Multidrug Resistance Transporters in the First Trimester Human Placenta. *J. Cell Mol. Med.* **2018**, *22*, 3652-3660.
62. Pavek, P.; Cerveny, L.; Svecova, L.; Brysch, M.; Libra, A.; Vrzal, R.; Nachtigal, P.; Staud, F.; Ulrichova, J.; Fendrich, Z.; Dvorak, Z. Examination of Glucocorticoid Receptor Alpha-Mediated Transcriptional Regulation of P-Glycoprotein, CYP3A4, and CYP2C9 genes in Placental Trophoblast Cell Lines. *Placenta* **2007**, *28*, 1004-1011.
63. Coles, L.D.; Lee, I.J.; Voulalas, P.J.; Eddington, N.D. Estradiol and Progesterone-Mediated Regulation of P-gp in P-gp Overexpressing Cells (NCI-ADR-RES) and Placental Cells (JAR). *Mol. Pharm.* **2009**, *6*, 1816-1825.
64. Evseenko, D.A.; Paxton, J.W.; Keelan, J.A. Independent Regulation of Apical and Basolateral Drug Transporter Expression and Function in Placental Trophoblasts by Cytokines, Steroids, and Growth Factors. *Drug Metab. Dispos.* **2007**, *35*, 595-601.
65. Kojovic, D.; Ghoneim, R.H.; Serghides, L.; Piquette-Miller, M. Role of HIV and Antiretroviral Therapy on the Expression of Placental Transporters in Women with HIV. *AAPS J.* **2020**, *22*, 138.
66. Dunk, C.E.; Pappas, J.J.; Lye, P.; Kibschull, M.; Javam, M.; Bloise, E.; Lye, S.J.; Szyf, M.; Matthews, S.G. P-Glycoprotein (P-gp)/ABCB1 Plays a Functional Role in Extravillous Trophoblast (EVT) Invasion and is Decreased in the Pre-Eclamptic Placenta. *J. Cell Mol. Med.* **2018**, *22*, 5378-5393.
67. Anger, G.J.; Cressman, A.M.; Piquette-Miller, M. Expression of ABC Efflux Transporters in Placenta from Women with Insulin-Managed Diabetes. *PLoS One* **2012**, *7*, e35027.
68. Zeng, Q.; Bai, M.; Li, C.; Lu, S.; Ma, Z.; Zhao, Y.; Zhou, H.; Jiang, H.; Sun, D.; Zheng, C. Multiple Drug Transporters Contribute to the Placental Transfer of Emtricitabine. *Antimicrob. Agents Chemother.* **2019**, *63*, e00199-19.
69. Feinshtein, V.; Holcberg, G.; Amash, A.; Erez, N.; Rubin, M.; Sheiner, E.; Polachek, H.; Ben-Zvi, Z. Nitrofurantoin Transport by Placental Choriocarcinoma JAr Cells: Involvement of BCRP, OATP2B1 and Other MDR Transporters. *Arch. Gynecol. Obstet.* **2010**, *281*, 1037-1044.
70. Gedeon, C.; Anger, G.; Piquette-Miller, M.; Koren, G. Breast Cancer Resistance Protein: Mediating the Trans-Placental Transfer of Glyburide Across the Human Placenta. *Placenta* **2008**, *29*, 39-43.
71. Pollex, E.; Lubetsky, A.; Koren, G. The Role of Placental Breast Cancer Resistance Protein in the Efflux of Glyburide Across the Human Placenta. *Placenta* **2008**, *29*, 743-747.
72. Gedeon, C.; Behravan, J.; Koren, G.; Piquette-Miller, M. Transport of Glyburide by Placental ABC Transporters: Implications in Fetal Drug Exposure. *Placenta* **2006**, *27*, 1096-1102.
73. Bircsak, K.M.; Gupta, V.; Yuen, P.Y.; Gorczyca, L.; Weinberger, B.I.; Vetrano, A.M.; Aleksunes, L.M. Genetic and Dietary Regulation of Glyburide Efflux by the Human Placental Breast Cancer Resistance Protein Transporter. *J. Pharmacol. Exp. Ther.* **2016**, *357*, 103-113.
74. Neradugomma, N.K.; Liao, M.Z.; Mao, Q. Buprenorphine, Norbuprenorphine, R-Methadone, and S-Methadone Upregulate BCRP/ABCG2 Expression by Activating Aryl Hydrocarbon Receptor in Human Placental Trophoblasts. *Mol. Pharmacol.* **2017**, *91*, 237-249.
75. Blazquez, A.G.; Briz, O.; Gonzalez-Sanchez, E.; Perez, M.J.; Ghanem, C.I.; Marin, J.J. The Effect of Acetaminophen on the Expression of BCRP in Trophoblast Cells Impairs the Placental Barrier to Bile Acids During Maternal Cholestasis. *Toxicol. Appl. Pharmacol.* **2014**, *277*, 77-85.
76. Feinshtein, V.; Erez, O.; Ben-Zvi, Z.; Eshkoli, T.; Sheizaf, B.; Sheiner, E.; Holcberg, G. Cannabidiol Enhances Xenobiotic Permeability Through the Human Placental Barrier by Direct Inhibition of Breast Cancer Resistance Protein: An Ex Vivo Study. *Am. J. Obstet. Gynecol.* **2013**, *209*, 573.e1-573.e15.

77. Grube, M.; Reuther, S.; Meyer zu Schwabedissen, H.; Köck, K.; Draber, K.; Ritter, C.A.; Fusch, C.; Jedlitschky, G.; Kroemer, H.K. Organic Anion Transporting Polypeptide 2B1 and Breast Cancer Resistance Protein Interact in the Transepithelial Transport of Steroid Sulfates in Human Placenta. *Drug Metab. Dispos.* **2007**, *35*, 30-35.
78. Zhou, L.; Naraharisetti, S.B.; Wang, H.; Unadkat, J.D.; Hebert, M.F.; Mao, Q. The Breast Cancer Resistance Protein (Bcrp1/Abcg2) Limits Fetal Distribution of Glyburide in the Pregnant Mouse: An Obstetric-Fetal Pharmacology Research Unit Network and University of Washington Specialized Center of Research Study. *Mol. Pharmacol.* **2008**, *73*, 949-959.
79. Blazquez, A.G.; Briz, O.; Romero, M.R.; Rosales, R.; Monte, M.J.; Vaquero, J.; Macias, R.I.; Cassio, D.; Marin, J.J. Characterization of the Role of ABCG2 as a Bile Acid Transporter in Liver and Placenta. *Mol. Pharmacol.* **2012**, *81*, 273-283.
80. Yeboah, D.; Sun, M.; Kingdom, J.; Baczyk, D.; Lye, S.J.; Matthews, S.G.; Gibb, W. Expression of Breast Cancer Resistance Protein (BCRP/ABCG2) in Human Placenta Throughout Gestation and At Term Before and After Labor. *Can. J. Physiol. Pharmacol.* **2006**, *84*, 1251-1258.
81. Petrovic, V.; Kojovic, D.; Cressman, A.; Piquette-Miller, M. Maternal Bacterial Infections Impact Expression of Drug Transporters in Human Placenta. *Int. Immunopharmacol.* **2015**, *26*, 349-356.
82. Sieppi, E.; Vähäkangas, K.; Rautio, A.; Ietta, F.; Paulesu, L.; Myllynen, P. The Xenoestrogens, Bisphenol A and Para-Nonylphenol, Decrease the Expression of the ABCG2 Transporter Protein in Human Term Placental Explant Cultures. *Mol. Cell. Endocrinol.* **2016**, *429*, 41-49.
83. Polachek, H.; Debotton, N.; Feinshtein, V.; Rubin, M.; Ben-Zvi, Z.; Holcberg, G.; Agbaria, R.; Dahan, A. The Role of Various Transporters in the Placental Uptake of Ofloxacin in an In Vitro Model of Human Villous Trophoblasts. *Drug Des. Devel. Ther.* **2018**, *12*, 4129-4138.
84. Wang, H.; Zhou, L.; Gupta, A.; Vethanayagam, R.R.; Zhang, Y.; Unadkat, J.D.; Mao, Q. Regulation of BCRP/ABCG2 Expression by Progesterone and 17Beta-Estradiol in Human Placental BeWo Cells. *Am. J. Physiol. Endocrinol. Metab.* **2006**, *290*, E798-E807.
85. Wang, H.; Unadkat, J.D.; Mao, Q. Hormonal Regulation of BCRP Expression in Human Placental BeWo Cells. *Pharm. Res.* **2008**, *25*, 444-452.
86. Wang, H.; Lee, E.W.; Zhou, L.; Leung, P.C.; Ross, D.D.; Unadkat, J.D.; Mao, Q. Progesterone Receptor (PR) Isoforms PRA and PRB Differentially Regulate Expression of the Breast Cancer Resistance Protein in Human Placental Choriocarcinoma BeWo Cells. *Mol. Pharmacol.* **2008**, *73*, 845-854.
87. Mason, C.W.; Lee, G.T.; Dong, Y.; Zhou, H.; He, L.; Weiner, C.P. Effect of Prostaglandin E2 on Multidrug Resistance Transporters in Human Placental Cells. *Drug Metab. Dispos.* **2014**, *42*, 2077-2086.
88. de Lima Moreira, F.; Melli, P.P.; Marques, M.P.; Rocha, A.; Nardotto, G.H.; Duarte, G.; Lanchote, V.L. P-Glycoprotein and Organic Anion Transporter Polypeptide 1B/Breast Cancer Resistance Protein Drug Transporter Activity in Pregnant Women Living With HIV. *J. Clin. Pharmacol.* **2023**, *63*, 219-227.
89. Kojovic, D.; Workewych, N.V.; Piquette-Miller, M. Role of Elevated SFLT-1 on the Regulation of Placental Transporters in Women with Pre-Eclampsia. *Clin. Transl. Sci.* **2020**, *13*, 580-588.
90. Evseenko, D.A.; Murthi, P.; Paxton, J.W.; Reid, G.; Emerald, B.S.; Mohankumar, K.M.; Lobie, P.E.; Brennecke, S.P.; Kalionis, B.; Keelan, J.A. The ABC Transporter BCRP/ABCG2 is a Placental Survival Factor, and Its Expression is Reduced in Idiopathic Human Fetal Growth Restriction. *FASEB J.* **2007**, *21*, 3592-3605.
91. Azzaroli, F.; Raspanti, M.E.; Simoni, P.; Montagnani, M.; Lisotti, A.; Cecinato, P.; Arena, R.; Simonazzi, G.; Farina, A.; Rizzo, N.; Mazzella, G. High Doses of Ursodeoxycholic Acid Up-Regulate the Expression of Placental Breast Cancer Resistance Protein in Patients Affected by Intrahepatic Cholestasis of Pregnancy. *PLoS One* **2013**, *8*, e64101.
92. Patel, P.; Weerasekera, N.; Hitchins, M.; Boyd, C.A.; Johnston, D.G.; Williamson, C. Semi Quantitative Expression Analysis of MDR3, FIC1, BSEP, OATP-A, OATP-C, OATP-D, OATP-E and NTCP Gene Transcripts in 1st and 3rd Trimester Human Placenta. *Placenta* **2003**, *24*, 39-44.
93. Keitel, V.; Vogt, C.; Häussinger, D.; Kubitz, R. Combined Mutations of Canalicular Transporter Proteins Cause Severe Intrahepatic Cholestasis of Pregnancy. *Gastroenterology* **2006**, *131*, 624-629.
94. Imperio, G.E.; Javam, M.; Lye, P.; Constantinof, A.; Dunk, C.E.; Reis, F.M.; Lye, S.J.; Gibb, W.; Matthews, S.G.; Ortiga-Carvalho, T.M.; Bloise, E. Gestational Age-Dependent Gene Expression Profiling

of ATP-Binding Cassette Transporters in the Healthy Human Placenta. *J. Cell Mol. Med.* **2019**, *23*, 610-618.

95. Song, X.; Vasilenko, A.; Chen, Y.; Valanejad, L.; Verma, R.; Yan, B.; Deng, R. Transcriptional Dynamics of Bile Salt Export Pump During Pregnancy: Mechanisms and Implications in Intrahepatic Cholestasis of Pregnancy. *Hepatology* **2014**, *60*, 1993-2007.
96. Gedeon, C.; Anger, G.; Lubetsky, A.; Miller, M.P.; Koren, G. Investigating the Potential Role of Multi-Drug Resistance Protein (MRP) Transporters in Fetal to Maternal Glyburide Efflux in the Human Placenta. *J. Obstet. Gynaecol.* **2008**, *28*, 485-489.
97. Vaidya, S.S.; Walsh, S.W.; Gerk, P.M. Formation and Efflux of ATP-Binding Cassette Transporter Substrate 2,4-Dinitrophenyl-S-Glutathione from Cultured Human Term Placental Villous Tissue Fragments. *Mol. Pharm.* **2009**, *6*, 1689-1702.
98. McColl, E.R.; Kwok, J.; Benowitz, N.L.; Patten, C.A.; Hughes, C.A.; Koller, K.R.; Flanagan, C.A.; Thomas, T.K.; Hiratsuka, V.Y.; Tyndale, R.F.; Piquette-Miller, M. The Effect of Tobacco Use on the Expression of Placental Transporters in Alaska Native Women. *Clin. Pharmacol. Ther.* **2023**, *113*, 634-642.
99. May, K.; Minarikova, V.; Linnemann, K.; Zygmunt, M.; Kroemer, H.K.; Fusch, C.; Siegmund, W. Role of the Multidrug Transporter Proteins ABCB1 and ABCC2 in the Diaplacental Transport of Talinolol in the Term Human Placenta. *Drug Metab. Dispos.* **2008**, *36*, 740-744.
100. Meyer zu Schwabedissen, H.E.; Jedlitschky, G.; Gratz, M.; Haenisch, S.; Linnemann, K.; Fusch, C.; Cascorbi, I.; Kroemer, H.K. Variable Expression of MRP2 (ABCC2) in Human Placenta: Influence of Gestational Age and Cellular Differentiation. *Drug Metab. Dispos.* **2005**, *33*, 896-904.
101. Liu, X.; Lai, H.; Xin, S.; Li, Z.; Zeng, X.; Nie, L.; Liang, Z.; Wu, M.; Zheng, J.; Zou, Y. Whole-Exome Sequencing Identifies Novel Mutations in ABC Transporter Genes Associated with Intrahepatic Cholestasis of Pregnancy Disease: A Case-Control Study. *BMC Pregnancy Childbirth* **2021**, *21*, 110.
102. Rao, Z.Z.; Zhang, X.W.; Ding, Y.L.; Yang, M.Y. miR-148a-Mediated Estrogen-Induced Cholestasis in Intrahepatic Cholestasis of Pregnancy: Role of PXR/MRP3. *PLoS One* **2017**, *12*, e0178702.
103. Chen, J.J.; Xiao, Z.J.; Meng, X.; Wang, Y.; Yu, M.K.; Huang, W.Q.; Sun, X.; Chen, H.; Duan, Y.G.; Jiang, X.; Wong, M.P.; Chan, H.C.; Zou, F.; Ruan, Y.C. MRP4 Sustains Wnt/β-Catenin Signaling for Pregnancy, Endometriosis and Endometrial Cancer. *Theranostics* **2019**, *9*, 5049-5064.
104. Meyer zu Schwabedissen, H.E.; Grube, M.; Heydrich, B.; Linnemann, K.; Fusch, C.; Kroemer, H.K.; Jedlitschky, G. Expression, Localization, and Function of MRP5 (ABCC5), a Transporter for Cyclic Nucleotides, in Human Placenta and Cultured Human Trophoblasts: Effects of Gestational Age and Cellular Differentiation. *Am. J. Pathol.* **2005**, *166*, 39-48.
105. Scott, H.; Martinelli, L.M.; Grynspan, D.; Bloise, E.; Connor, K.L. Preterm Birth Associates with Increased Placental Expression of MDR Transporters Irrespective of Prepregnancy BMI. *J. Clin. Endocrinol. Metab.* **2022**, *107*, 1140-1158.
106. Berveiller, P.; Mir, O.; Degrelle, S.A.; Tsatsaris, V.; Selleret, L.; Guibourdenche, J.; Evain-Brion, D.; Fournier, T.; Gil, S. Chemotherapy in Pregnancy: Exploratory Study of the Effects of Paclitaxel on the Expression of Placental Drug Transporters. *Invest. New Drugs* **2019**, *37*, 1075-1085.
107. Fokina, V.M.; Patrikeeva, S.; Wang, X.M.; Noguchi, S.; Tomi, M.; König, J.; Ahmed, M.S.; Nanovskaya, T. Role of Uptake Transporters OAT4, OATP2A1, and OATP1A2 in Human Placental Bio-Disposition of Pravastatin. *J. Pharm. Sci.* **2022**, *111*, 505-516.
108. Wang, H.; Yan, Z.; Dong, M.; Zhu, X.; Wang, H.; Wang, Z. Alteration in Placental Expression of Bile Acids Transporters OATP1A2, OATP1B1, OATP1B3 in Intrahepatic Cholestasis of Pregnancy. *Arch. Gynecol. Obstet.* **2012**, *285*, 1535-1540.
109. Nabekura, T.; Kawasaki, T.; Kamiya, Y.; Uwai, Y. Effects of Antiviral Drugs on Organic Anion Transport in Human Placental BeWo Cells. *Antimicrob. Agents Chemother.* **2015**, *59*, 7666-7670.
110. Lofthouse, E.M.; Cleal, J.K.; Hudson, G.; Lewis, R.M.; Sengers, B.G. Glibenclamide Transfer Across the Perfused Human Placenta is Determined by Albumin Binding Not Transporter Activity. *Eur. J. Pharm. Sci.* **2020**, *152*, 105436.
111. Hasegawa, N.; Furugen, A.; Ono, K.; Koishikawa, M.; Miyazawa, Y.; Nishimura, A.; Umazume, T.; Narumi, K.; Kobayashi, M.; Iseki, K. Cellular Uptake Properties of Lamotrigine in Human Placental Cell

Lines: Investigation of Involvement of Organic Cation Transporters (SLC22A1-5). *Drug Metab. Pharmacokinet.* **2020**, *35*, 266-273.
112. Wessler, I.; Roth, E.; Deutsch, C.; Brockerhoff, P.; Bittinger, F.; Kirkpatrick, C.J.; Kilbinger, H. Release of Non-Neuronal Acetylcholine from the Isolated Human Placenta is Mediated by Organic Cation Transporters. *Br. J. Pharmacol.* **2001**, *134*, 951-956.
113. Bergagnini-Kolev, M.C.; Hebert, M.F.; Easterling, T.R.; Lin, Y.S. Pregnancy Increases the Renal Secretion of N1-methylnicotinamide, an Endogenous Probe for Renal Cation Transporters, in Patients Prescribed Metformin. *Drug Metab. Dispos.* **2017**, *45*, 325-329.
114. Ahmadimoghaddam, D.; Zemankova L.; Nachtigal, P.; Dolezelova, E.; Neumanova, Z.; Cerveny, L.; Ceckova, M.; Kacerovsky, M.; Micuda, S.; Staud, F. Organic Cation Transporter 3 (OCT3/SLC22A3) and Multidrug and Toxin Extrusion 1 (MATE1/SLC47A1) Transporter in the Placenta and Fetal Tissues: Expression Profile and Fetus Protective Role at Different Stages of Gestation. *Biol. Reprod.* **2013**, *88*, 55.
115. Lee, N.; Hebert, M.F.; Wagner, D.J.; Easterling, T.R.; Liang, C.J.; Rice, K.; Wang, J. Organic Cation Transporter 3 Facilitates Fetal Exposure to Metformin during Pregnancy. *Mol Pharmacol.* **2018**, *94*, 1125-1131.
116. Lee, N.; Hebert, M.F.; Prasad, B.; Easterling, T.R.; Kelly, E.J.; Unadkat, J.D.; Wang, J. Effect of Gestational Age on mRNA and Protein Expression of Polyspecific Organic Cation Transporters During Pregnancy. *Drug Metab. Dispos.* **2013**, *41*, 2225-2232.
117. Sata, R.; Ohtani, H.; Tsujimoto, M.; Murakami, H.; Koyabu, N.; Nakamura, T.; Uchiumi, T.; Kuwano, M.; Nagata, H.; Tsukimori, K.; Nakano, H.; Sawada, Y. Functional Analysis of Organic Cation Transporter 3 Expressed in Human Placenta. *J. Pharmacol. Exp. Ther.* **2005**, *315*, 888-895.
118. Wu, S.P.; Shyu, M.K.; Liou, H.H.; Gau, C.S.; Lin, C.J. Interaction Between Anticonvulsants and Human Placental Carnitine Transporter. *Epilepsia.* **2004**, *45*, 204-210.
119. Grube, M.; Meyer zu Schwabedissen, H.; Draber, K.; Präger, D.; Möritz, K.U.; Linnemann, K.; Fusch, C.; Jedlitschky, G.; Kroemer, H.K. Expression, Localization, and Function of the Carnitine transporter Octn2 (Slc22a5) in Human Placenta. *Drug Metab. Dispos.* **2005**, *33*, 31-37.
120. Rytting, E.; Audus, K.L. Novel Organic Cation Transporter 2-Mediated Carnitine Uptake in Placental Choriocarcinoma (BeWo) Cells. *J. Pharmacol. Exp. Ther.* **2005**, *312*, 192-198.
121. Hirano, T.; Yasuda, S.; Osaka, Y.; Asari, M.; Kobayashi, M.; Itagaki, S.; Iseki, K. The Inhibitory Effects of Fluoroquinolones on L-Carnitine Transport in Placental Cell Line BeWo. *Int. J. Pharm.* **2008**, *351*, 113-118.
122. Peng, J.; Ladumor, M.K.; Unadkat, J.D. Prediction of Pregnancy-Induced Changes in Secretory and Total Renal Clearance of Drugs Transported by Organic Anion Transporters. *Drug Metab. Dispos.* **2021**, *49*, 929-937.
123. Ma, Z.; Lu, S.; Sun, D.; Bai, M.; Jiang, T.; Lin, N.; Zhou, H.; Zeng, S.; Jiang, H. Roles of Organic Anion Transporter 2 and Equilibrative Nucleoside Transporter 1 in Hepatic Disposition and Antiviral Activity of Entecavir During Non-Pregnancy and Pregnancy. *Br. J. Pharmacol.* **2019**, *176*, 3236-3249.
124. Noguchi, S.; Nishimura, T.; Fujibayashi, A.; Maruyama, T.; Tomi, M.; Nakashima, E. Organic Anion Transporter 4-Mediated Transport of Olmesartan at Basal Plasma Membrane of Human Placental Barrier. *J. Pharm. Sci.* **2015**, *104*, 3128-3135.
125. Cha, S.H.; Sekine, T.; Kusuhara, H.; Yu, E.; Kim, J.Y.; Kim, D.K.; Sugiyama, Y.; Kanai, Y.; Endou, H. Molecular Cloning and Characterization of Multispecific Organic Anion Transporter 4 Expressed in the Placenta. *J. Biol. Chem.* **2000**, *275*, 4507-4512.
126. Zhou, F.; Hong, M.; You, G. Regulation of Human Organic Anion Transporter 4 by Progesterone and Protein Kinase C in Human Placental BeWo Cells. *Am. J. Physiol. Endocrinol. Metab.* **2007**, *293*, E57-E61.
127. Zhang, Y.; Chen, Y.; Dai, B.; Bai, M.; Lu, S.; Lin, N.; Zhou, H.; Jiang, H. Bilirubin Reduces the Uptake of Estrogen Precursors and the Followed Synthesis of Estradiol in Human Placental Syncytiotrophoblasts via Inhibition and Downregulation of Organic Anion Transporter 4. *Drug Metab. Dispos.* **2022**, *50*, 341-350.
128. Jiraskova, L.; Cerveny, L.; Karbanova, S.; Ptackova, Z.; Staud, F. Expression of Concentrative Nucleoside Transporters (SLC28A) in the Human Placenta: Effects of Gestation Age and Prototype Differentiation-Affecting Agents. *Mol. Pharm.* **2018**, *15*, 2732-2741.

129. Govindarajan, R.; Bakken, A.H.; Hudkins, K.L.; Lai, Y.; Casado, F.J.; Pastor-Anglada, M.; Tse, C.M.; Hayashi, J.; Unadkat, J.D. In Situ Hybridization and Immunolocalization of Concentrative and Equilibrative Nucleoside Transporters in the Human Intestine, Liver, Kidneys, and Placenta. *Am. J. Physiol. Regul. Integr. Comp. Physiol.* **2007**, *293*, R1809-R1822.
130. Nishimura, T.; Sano, Y.; Takahashi, Y.; Noguchi, S.; Uchida, Y.; Takagi, A.; Tanaka, T.; Katakura, S.; Nakashima, E.; Tachikawa, M.; Maruyama, T.; Terasaki, T.; Tomi, M. Quantification of ENT1 and ENT2 Proteins at the Placental Barrier and Contribution of These Transporters to Ribavirin Uptake. *J. Pharm. Sci.* **2019**, *108*, 3917-3922.
131. Cerveny, L.; Ptackova, Z.; Ceckova, M.; Karahoda, R.; Karbanova, S.; Jiraskova, L.; Greenwood, S.L.; Glazier, J.D.; Staud, F. Equilibrative Nucleoside Transporter 1 (ENT1, SLC29A1) Facilitates Transfer of the Antiretroviral Drug Abacavir across the Placenta. *Drug Metab. Dispos.* **2018**, *46*, 1817-1826.
132. Karbanova, S.; Cerveny, L.; Jiraskova, L.; Karahoda, R.; Ceckova, M.; Ptackova, Z.; Staud, F. Transport of Ribavirin Across the Rat and Human Placental Barrier: Roles of Nucleoside and ATP-Binding Cassette Drug Efflux Transporters. *Biochem. Pharmacol.* **2019**, *163*, 60-70.
133. Cerveny, L.; Karbanova, S.; Karahoda, R.; Horackova, H.; Jiraskova, L.; Ali, M.N.; Staud, F. Assessment of the Role of Nucleoside Transporters, P-Glycoprotein, Breast Cancer Resistance Protein, and Multidrug Resistance-Associated Protein 2 in the Placental Transport of Entecavir Using In Vitro, Ex Vivo, and In Situ Methods. *Toxicol. Appl. Pharmacol.* **2023**, *463*, 116427.
134. Griffiths, M.; Yao, S.Y.; Abidi, F.; Phillips, S.E.; Cass, C.E.; Young, J.D.; Baldwin, S.A. Molecular Cloning and Characterization of a Nitrobenzylthioinosine-Insensitive (Ei) Equilibrative Nucleoside Transporter from Human Placenta. *Biochem. J.* **1997**, *328*, 739-743.
135. Escudero, C.; Casanello, P.; Sobrevia, L. Human Equilibrative Nucleoside Transporters 1 and 2 May Be Differentially Modulated by A2B Adenosine Receptors in Placenta Microvascular Endothelial Cells from Pre-Eclampsia. *Placenta* **2008**, *29*, 816-825.
136. Pfeifer, E.; Parrott, J.; Lee, G.T.; Domalakes, E.; Zhou, H.; He, L.; Mason, C.W. Regulation of Human Placental Drug Transporters in HCV Infection and Their Influence on Direct Acting Antiviral Medications. *Placenta* **2018**, *69*, 32-39.
137. Salomón, C.; Westermeier, F.; Puebla, C.; Arroyo, P.; Guzmán-Gutiérrez, E.; Pardo, F.; Leiva, A.; Casanello, P.; Sobrevia, L. Gestational Diabetes Reduces Adenosine Transport in Human Placental Microvascular Endothelium, an Effect Reversed by Insulin. *PLoS One* **2012**, *7*, e40578.
138. Ceckova, M.; Reznicek, J.; Ptackova, Z.; Cerveny, L.; Müller, F.; Kacerovsky, M.; Fromm, M.F.; Glazier, J.D.; Staud, F. Role of ABC and Solute Carrier Transporters in the Placental Transport of Lamivudine. *Antimicrob. Agents Chemother.* **2016**, *60*, 5563-5572.
139. Landor, M.; Rubinstein, A.; Kim, A.; Calvelli, T.; Mizrachi, Y. Receptor-Mediated Maternofetal Transfer of Immunoglobulins. Inhibition of Transport of Anti-HIV-1 Immunoglobulin by Generic Immunoglobulins in the In Vitro Perfused Placenta. *Int. Arch. Allergy Immunol.* **1998**, *115*, 203-209.
140. Ellinger, I.; Schwab, M.; Stefanescu, A.; Hunziker, W.; Fuchs, R. IgG Transport Across Trophoblast-Derived BeWo Cells: A Model System to Study IgG Transport in the Placenta. *Eur. J. Immunol.* **1999**, *29*, 733-744.
141. Einarsdottir, H.K.; Stapleton, N.M.; Scherjon, S.; Andersen, J.T.; Rispens, T.; van der Schoot, C.E.; Vidarsson, G. On the Perplexingly Low Rate of Transport of IgG2 Across the Human Placenta. *PLoS One* **2014**, *9*, e108319.
142. Palfi, M.; Selbing, A. Placental Transport of Maternal Immunoglobulin G. *Am. J. Reprod. Immunol.* **1998**, *39*, 24-26.

Additional References

143. Reproductive Development & Function of the Female Reproductive System. In *Ganong's Review of Medical Physiology*, 26th ed.; Barrett, K.E., Barman, S.M., Brooks, H.L., Yuan, J.J., Eds.; McGraw Hill: New York, USA, 2019; ISBN 978-126-012-240-4.
144. Rouguieg, K.; Picard, N.; Sauvage, F.L.; Gaulier, J.M.; Marquet, P. Contribution of the Different UDP-Glucuronosyltransferase (UGT) Isoforms to Buprenorphine and Norbuprenorphine Metabolism and Relationship with the Main UGT Polymorphisms in a Bank of Human Liver Microsomes. *Drug Metab. Dispos.* **2010**, *38*, 40-45.
145. Kunze, A.; Huwyler, J.; Camenisch, G.; Poller, B. Prediction of Organic Anion-Transporting Polypeptide 1B1- and 1B3-Mediated Hepatic Uptake of Statins Based on Transporter Protein Expression and Activity Data. *Drug Metab. Dispos.* **2014**, *42*, 1514-1521.
146. Kitamura, S.; Maeda, K.; Wang, Y.; Sugiyama, Y. Involvement of Multiple Transporters in the Hepatobiliary Transport of Rosuvastatin. *Drug Metab. Dispos.* **2008**, *36*, 2014-2023.
147. Mathialagan, S.; Feng, B.; Rodrigues, A.D.; Varma, M.V. Drug-Drug Interactions Involving Renal OCT2/MATE Transporters: Clinical Risk Assessment May Require Endogenous Biomarker-Informed Approach. *Clin. Pharmacol. Ther.* **2021**, *110*, 855-859.
148. Koepsell, H.; Lips, K.; Volk, C. Polyspecific Organic Cation Transporters: Structure, Function, Physiological Roles, and Biopharmaceutical Implications. *Pharm. Res.* **2007**, *24*, 1227-1251.
149. König, J.; Zolk, O.; Singer, K.; Hoffmann, C.; Fromm, M.F. Double-Transfected MDCK Cells Expressing Human OCT1/MATE1 or OCT2/MATE1: Determinants of Uptake and Transcellular Translocation of Organic Cations. *Br. J. Pharmacol.* **2011**, *163*, 546–555.
150. Pillai, V.C.; Shah, M.; Rytting, E.; Nanovskaya, T.N.; Wang, X.; Clark, S.M.; Ahmed, M.S.; Hankins, G.D.; Caritis, S.N.; Venkataramanan, R. Prediction of Maternal and Fetal Pharmacokinetics of Indomethacin in Pregnancy. *Br. J. Clin. Pharmacol.* **2022**, *88*, 271-281.
151. van Hoogdalem, M.W.; Wexelblatt, S.L.; Akinbi, H.T.; Vinks, A.A.; Mizuno, T. A Review of Pregnancy-Induced Changes in Opioid Pharmacokinetics, Placental Transfer, and Fetal Exposure: Towards Fetomaternal Physiologically-Based Pharmacokinetic Modeling to Improve the Treatment of Neonatal Opioid Withdrawal Syndrome. *Pharmacol. Ther.* **2022**, *234*, 108045.
152. Liao, M.Z.; Flood-Nichols, S.K.; Ahmed, M.; Clark, S.; Hankins, G.D.; Caritis, S.; Venkataramanan, R.; Haas, D.; Quinney, S.K.; Haneline, L.S.; et al. Effects of Pregnancy on the Pharmacokinetics of Metformin. *Drug Metab. Dispos.* **2020**, *48*, 264-271.
